# The peptide Acein promotes dopamine secretion through *clec-126* to extend the lifespan of elderly *C. elegans*

**DOI:** 10.18632/aging.205150

**Published:** 2023-12-27

**Authors:** Jiaqi Wang, Dong Wang, Sarra Setrerrahmane, Jean Martinez, Han-Mei Xu

**Affiliations:** 1 Synthetic Peptide Drug Discovery and Evaluation Engineering Research Center, China Pharmaceutical University, Nanjing 211198, China; 2 Institut des Biomolécules Max Mousseron (IBMM), Université de Montpellier, CNRS, ENSCM, Pôle Chimie Balard Recherche, Montpellier cedex 5 34293, France; 3 Nanjing ANJI Biotechnology Co., Ltd., Nanjing 210033, China

**Keywords:** aging, Acein, *Caenorhabditis elegans*, clec-126, dopamine

## Abstract

Dopamine plays a crucial role in regulating brain activity and movement and modulating human behavior, cognition and mood. Regulating dopamine signaling may improve cognitive abilities and physical functions during aging. Acein, a nonapeptide of sequence H-Pro-Pro-Thr-Thr-Thr-Lys-Phe-Ala-Ala-OH is able to stimulate dopamine secretion in the brain. By using genetic editing and lifespan investigation in *C. elegans*, we showed that the lack of the C-type lectin domain-containing protein *clec-126* significantly suppressed the aging phenotype and prolonged lifespan, while overexpression of *clec-126* promoted aging-related phenotypes and accelerated the aging process. We examined the aging phenotype of *C. elegans* and showed that Acein could induce a decrease in *clec-126* expression, prolonging the lifespan of aged *C. elegans*. The mechanism proceeds through the Acein-induced stimulation of dopamine secretion that ameliorates motor function decline and extends the healthy lifespan of aged *C. elegans*. In addition, we also observed an increase in brood number. Our study has shown that Acein regulates dopamine secretion and has good antiaging activity by decreasing *clec-126* expression.

## INTRODUCTION

Dopamine signaling is associated with cognitive performance and physical function during aging [1]. As a neurotransmitter, dopamine is widely present in animals, plants and microorganisms, and it can regulate a variety of physiological functions including learning and memory, motor behavior and reward decision-making behavior of the body [2]. Studies have shown that dopamine plays an important role in Parkinson’s disease (PD) [3]. Since the motor symptoms of PD are caused by the death of dopamine-releasing nerve cells from the substantia nigra pars compacta (SNc), patients with PD cannot secrete dopamine. On the other hand, the reduction or disappearance of the substantia nigra in the brain affects the transport of dopamine in the striatum, which regulates brain movements [4], and accelerates the aging process in elderly individuals. Increasing dopamine secretion either through transgenic or pharmacological strategies can slow aging and age-related diseases [5–7], and is also able to increase the learning ability and task performance in some older adults to younger adult levels [8, 9]. Dopamine content is generally age-dependent [10], and the older is the age, the faster is the clearance of dopamine [11]. This age-related decline in the dopamine system is prevalent in various animal models such as *Drosophila*, *Caenorhabditis elegans* (*C. elegans*), mice, rats, and monkeys, indicating that degeneration of the dopamine system function may have a conservative molecular mechanism [12]. However, very few studies have identified dopamine contents as a key to regulating aging, and investigating the relationship and the mechanism between dopamine and aging is interesting.

Acein, a nonapeptide of sequence H-Pro-Pro-Thr-Thr-Thr-Lys-Phe-Ala-Ala-OH stimulates dopamine release in the brain [13] by binding with high affinity to the striatal bound-membrane angiotensin converting enzyme (ACE) of rodents [13, 14]. Further structure-activity relationship studies revealed that the C-terminal pentapeptide of Acein H-Thr-Lys-Phe-Ala-Ala-OH is the smallest fragment exhibiting full biological activity [14, 15].

In this study we investigated the effect of Acein on lifespan, using the classic *C. elegans* aging organism model, in addition we identified the gene related to aging process and its relation with dopamine release.

## RESULTS

### Acein regulates aging by promoting dopamine release

**Figure 1:**
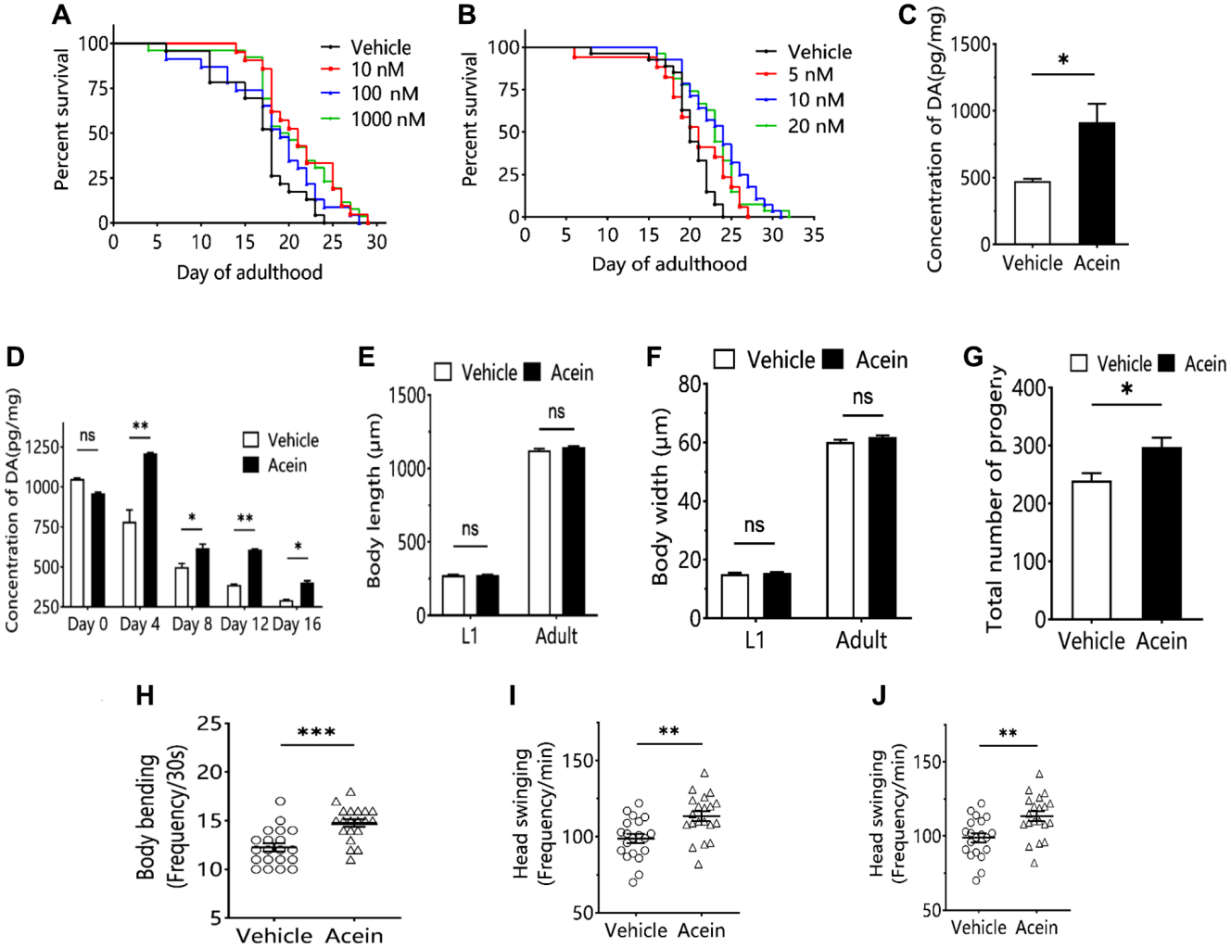
**Acein regulates aging by influencing dopamine level.** (**A**) Survival curve of 10 nM, 100 nM and 1000 nM Acein groups. (**B**) Survival curve of 5 nM, 10 nM and 20 nM Acein groups. (**C**) Dopamine content in *C.elegans* after 48 h of 10 nM Acein treatment. (**D**) Change of dopamine level in *C. elegans* with age after treatment with 10 nM Acein. (**E**) *C.elegans* body length after treatment with 10 nM Acein. (**F**) *C. elegans* body width after treatment with 10 nM Acein. (**G**) *C.elegans* progeny number after treatment with 10 nM Acein. (**H**) Body bending detection of *C.elegans* after treatment with 10 nM Acein. (**I**) Head swing detection of *C.elegans* after treatment with 10 nM Acein. (**J**) Pharyngeal pumping detection of *C.elegans*. Compared with the vehicle group. ^ns^*p* > 0.05, ^*^p < 0.05, ^**^p < 0.01, ^***^p < 0.001.

The effect of Acein on *C. elegans* longevity was investigated at different concentration. Acein showed the best antiaging activity at a 10 nM concentration, extending the mean lifespan by 25.66% and the median lifespan by 20.00% (Figure 1A, 1B). Forty-eight hours after administration of 10 nM Acein, dopamine secretion in *C. elegans* was increased by a factor of approximately 2 (Figure 1C). In contrast, 10 nM Acein did not affect the growth and development of L1-stage larvae and adults of *C. elegans* (Figure 1E, 1F). However, 10 nM Acein significantly increased the number of broods and improved the reproductive ability of *C. elegans* (Figure 1G). Acein was also able to improve the fitness of *C. elegans*, including body flexion, head swing and pharyngeal pumping frequency (Figure 1H–1J).

### Transcriptome sequencing revealed *clec-126*, a key gene for aging regulation in *C. elegans* by Acein

**Figure 2:**
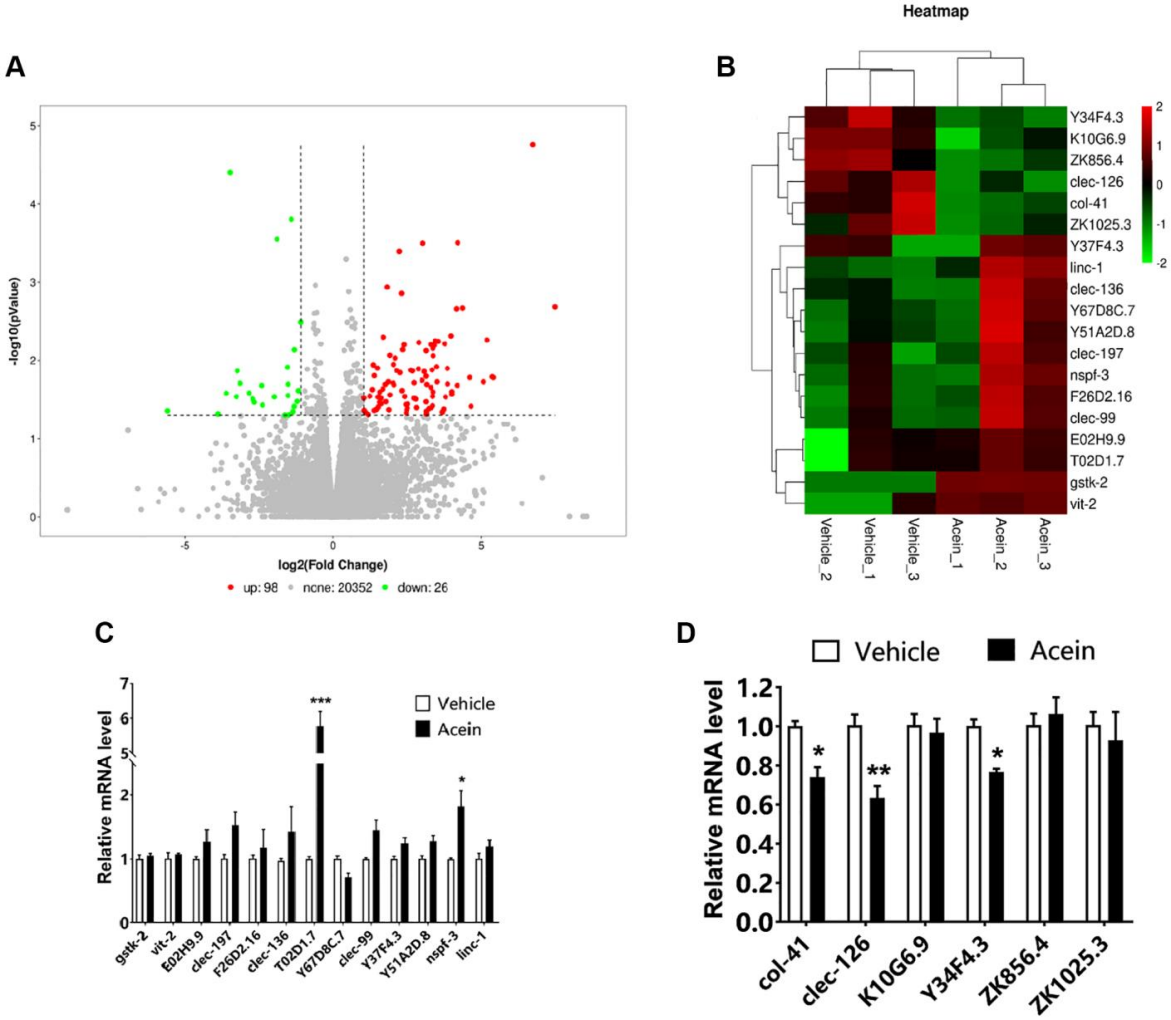
**Transcriptome sequencing revealed *clec-126*, a key gene for the anti-aging activity exerted by Acein.** (**A**) Differential gene scatter diagram, a total of 98 genes were up-regulated (red) and 26 genes were down-regulated (green). (**B**) Selected differential gene heat map, the screening criteria were FC ≥ 2 or FC ≤ 0.5, *P* value ≤ 0.05, and the reproducibility of the parallel group was good. (**C**) qPCR validation results of 13 up-regulated genes. (**D**) qPCR validation results of 6 down-regulated genes. Compared with the vehicle group, ^*^p < 0.05, ^**^p < 0.01, ^***^p < 0.001.

Transcriptome sequencing of *C. elegans* tissue samples showed that 98 genes were up-regulated and 26 genes were down-regulated in the 10 nM Acein-treated group (Figure 2A). Further screening was then performed based on HR fold difference and significance level *P* values (FC ≥ 2 or FC ≤ 0.5 (absolute logFC ≥ 1), *P* value ≤ 0.05) and good reproducibility between parallel groups. A total of 13 up-regulated genes and 6 down-regulated genes were identified (Figure 2B). qPCR verification showed that Acein could significantly upregulate *T02D1.7* and *nspf-3* and downregulate the expression of *col-41*, *clec-126* and *Y34F4.3*. The most significant effect was observed for *T02D1.7* on the upside and for *clec-126* on the downside (Figure 2C, 2D). Genetic information screening methods indicated that *T02D1.7* corresponded to an uncharacterized protein, while the C-type lectin domain protein encoded by *clec-126* has carbohydrate binding activity (Supplementary Tables 1, 2).

### Silencing *clec-126* can increase dopamine-induced Acein and prolong the lifespan of elderly *C. elegans*

**Figure 3:**
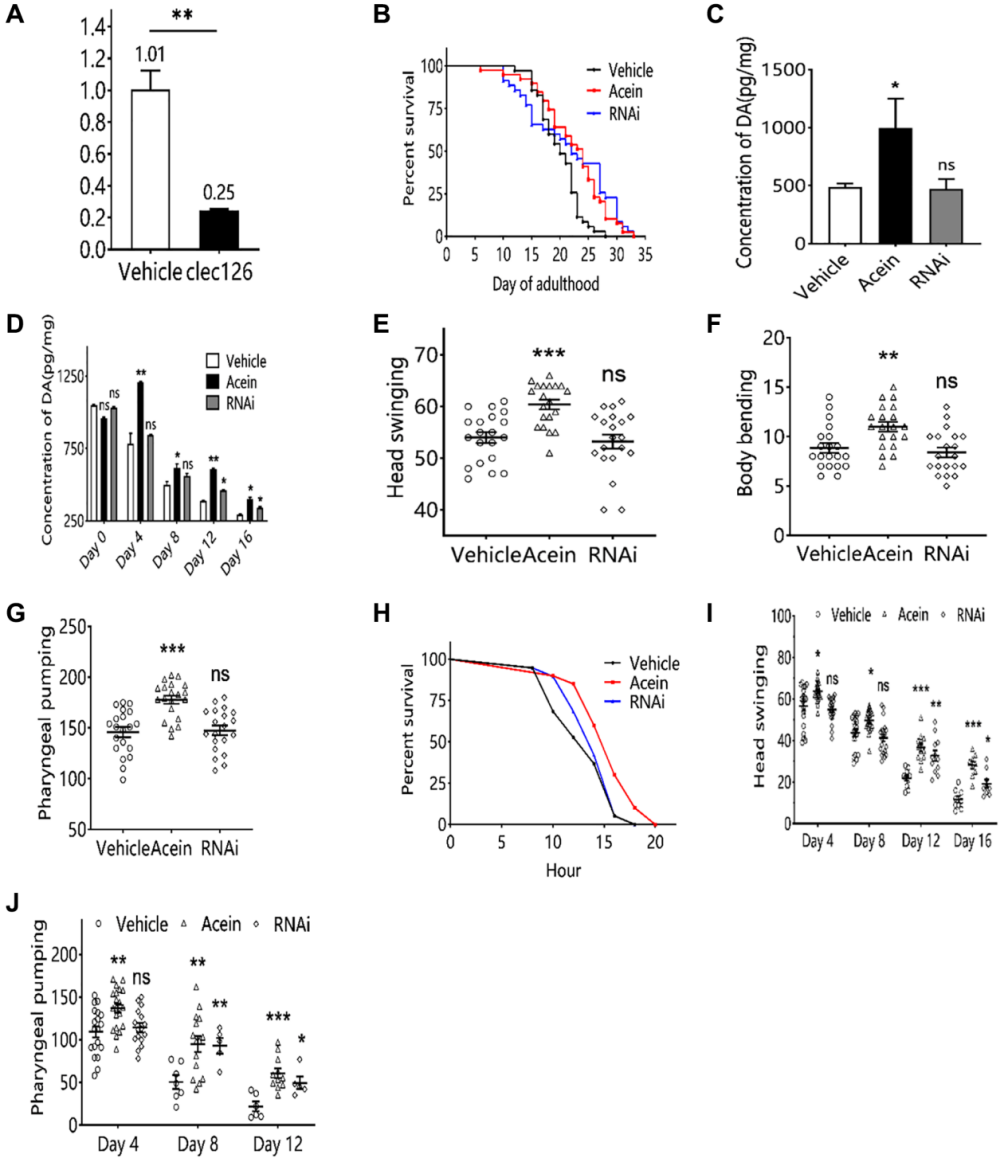
**Aging-associated phenotypic assay results in *C. elegans* with clec-126-RNAi.** (**A**) qPCR validation results of clec-126-RNAi *C. elegans*. (**B**) Lifespan experiments for clec-126-RNAi *C. elegans*. (**C**) Dopamine changes in *C. elegans* at 48 h. (**D**) Changes in dopamine levels in *C. elegans* with age. (**E**) Head swing in clec-126-RNAi *C. elegans*. (**F**) Body bending in clec-126-RNAi *C. elegans*. (**G**) Detection results of pharyngeal pumping in clec-126-RNAi *C. elegans*. (**H**) Survival curve on Day 8 in clec-126-RNAi *C. elegans* group in a thermostressed environment at 35°C. (**I**) Head swing frequency in clec-126-RNAi *C. elegans* of different age groups. (**J**) Pharyngeal pumping frequency in clec-126-RNAi *C. elegans* of different age groups. Compared with the vehicle group, ^ns^*p* > 0.05, ^*^p < 0.05, ^**^p < 0.01, ^***^p < 0.001.

Low *clec-126* expression in clec-126-RNAi *C. elegans* was verified by qPCR (Figure 3A). Lifespan assays showed that the lifespan of clec-126-RNAi *C. elegans* was significantly higher, with a maximum increase of 31.19% in mean lifespan and 41.18% in median lifespan (Figure 3B). Subsequently, we investigated whether silencing *clec-126* in *C. elegans* affected dopamine secretion levels. The results showed that silencing clec-126-RNAi could delay the decrease in dopamine content and increase dopamine levels in elderly *C. elegans* particularly at day 4 (Figure 3C, 3D). Because dopamine may affect the exercise capacity of *C. elegans*, the frequency of body bending, head swing and pharyngeal pumping was examined. The results showed that clec-126-RNAi did not affect the frequency of body bending, head swing or pharyngeal pumping in *C. elegans* (Figure 3E–3G). Stress capacity can reflect the body’s resistance to external stress, [16, 17] which is closely related to a healthy lifespan. The results showed that clec-126-RNAi could not significantly affect the survival of *C. elegans* under heat stress (Figure 3H). With age increasing, the motility of the body gradually decreased, which greatly affected the healthy lifespan and reduced the quality of life of the elderly population, so the head swing and pharyngeal pumping of clec-126-RNAi *C. elegans* at different ages were measured. The results showed that clec-126-RNAi could significantly slow the decline in head swing ability and pharyngeal pump ability of *C. elegans* (Figure 3I, 3J).

### Overexpression of *clec-126* in *C. elegans* reduces dopamine in the elderly and shortens their lifespan

**Figure 4:**
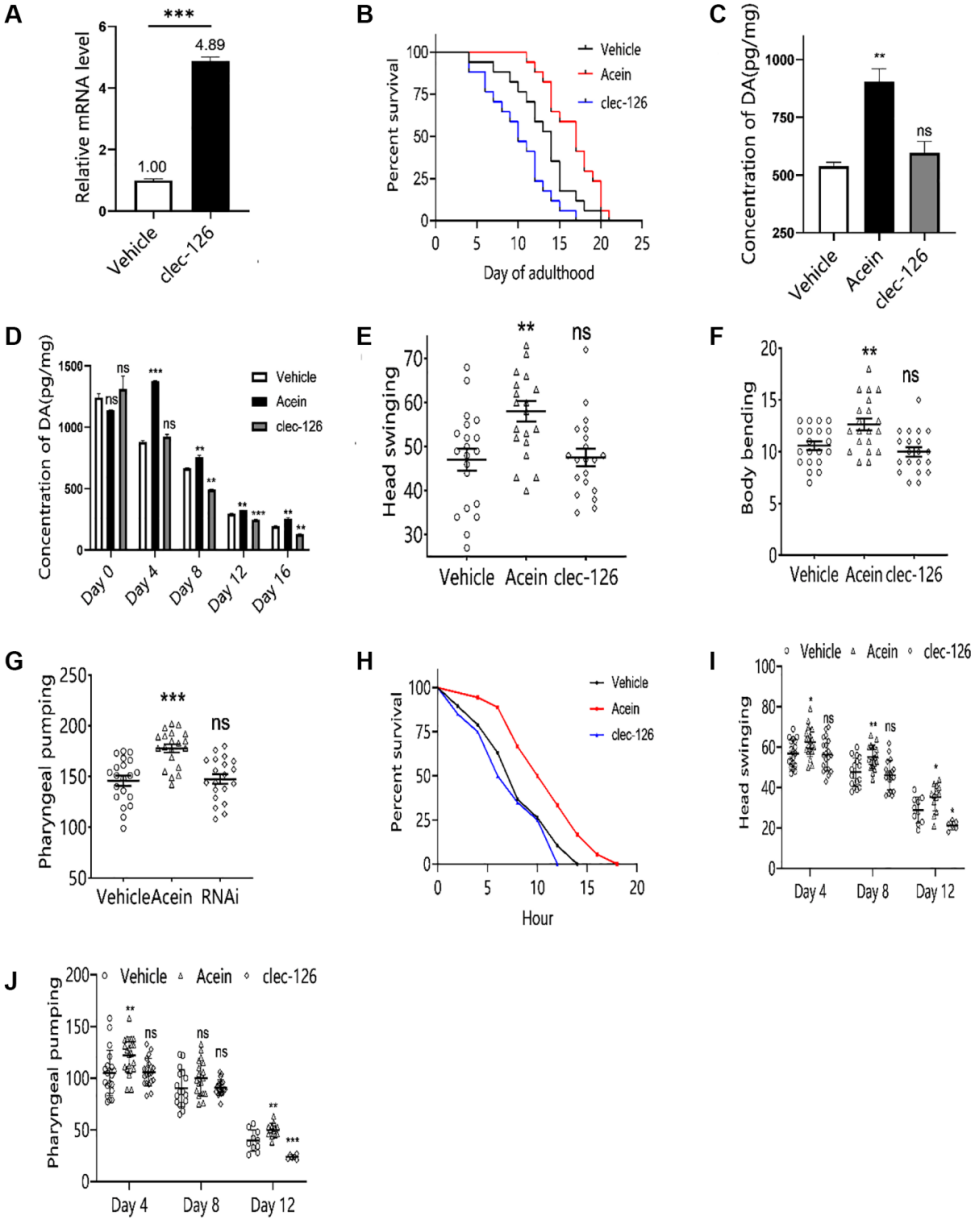
**Aging-associated phenotypic assay results in *C. elegans* overexpressing *clec-126*.** (**A**) qPCR validation results of *C. elegans* overexpressing *clec-126*. (**B**) Lifespan experiments of *C. elegans* overexpressing *clec-126*. (**C**) Dopamine changes in *C. elegans* at 48 h. (**D**) Changes in dopamine levels in *C. elegans* with age. (**E**) Head swing in *C. elegans* overexpressing *clec-126*. (**F**) Body bending in *C. elegans* overexpressing *clec-126*. (**G**) Pharyngeal pumping in *C. elegans* overexpressing *clec-126*. (**H**) Survival curve of Day 8 *C. elegans* overexpressing *clec-126* in a thermostressed environment at 35°C. (**I**) Head swing in *C. elegans* overexpressing *clec-126* of different age groups. (**J**) Detection results of pharyngeal pumping frequency in *C. elegans* overexpressing *clec-126* of different age groups. Compared with the vehicle group. ^ns^*p* > 0.05, ^*^p < 0.05, ^**^p < 0.01, ^***^p < 0.001.

Successful overexpression of *clec-126* in *C. elegans* was verified by qPCR technology (Figure 4A). The results showed that overexpression of *clec-126* significantly shortened the lifespan of *C. elegans*, with a maximum mean reduction of 23.96% and a median reduction of 33.33% (Figure 4B). With age, the dopamine content of *C. elegans* gradually decreased. *Clec-126* overexpression did not affect dopamine levels (*p* > 0.05) (Figure 4C) but accelerated its breakdown in aged *C. elegans* and significantly reduced its content in middle-aged *C. elegans* (*p* < 0.05) (Figure 4D), thereby accelerating aging. Motor behavior results showed that overexpression of *clec-126* in *C. elegans* had no significant effect on the head swing, body flexion and pharyngeal pumping frequency, suggesting that overexpression of *clec-126* did not affect the exercise capacity of *C. elegans* (Figure 4E–4G). However, overexpression of *clec-126* in aged *C. elegans* significantly accelerated the decline in head swing and pharyngeal pumping ability (Figure 4I, 4J). On the other hand, overexpression of *clec-126* had no significant effect on the survival time under heat stress but shortened the overall survival time of *C. elegans* at 35°C (Figure 4H).

### Interaction between *clec-126* and other differentially expressed genes

**Figure 5:**
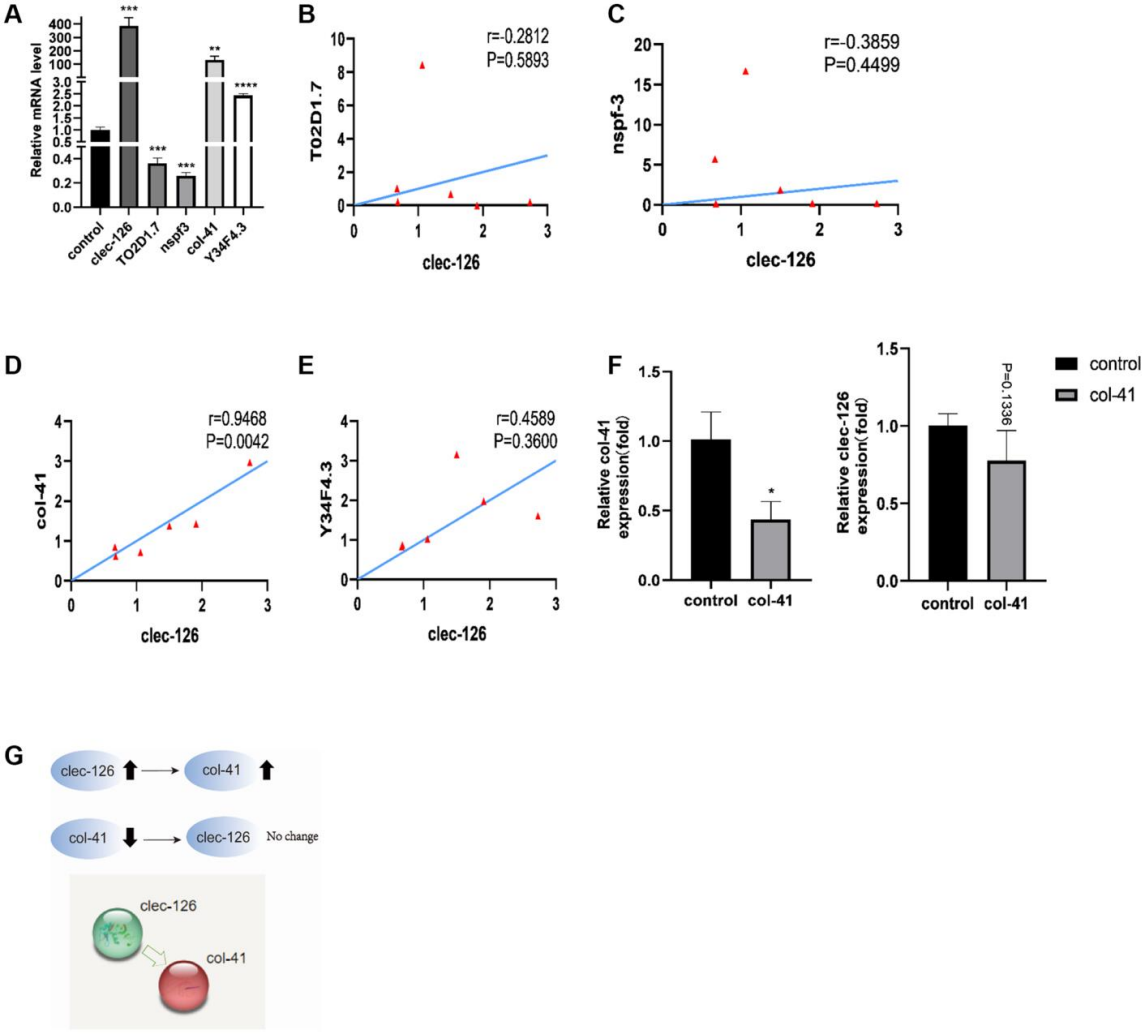
**Interaction of *clec-126* with four differentially expressed genes, *T02D1.7*, *nspf-3*, *col-41* and *Y34F4.3*.** (**A**) Differential expression of *T02D1.7*, *nspf-3*, *col-41* and *Y34F4.3* in overexpressing *clec-126 C. elegans*. (**B**–**E**) Correlation analysis between *clec-126* and *T02D1.7*, *nspf-3*, *col-41* and *Y34F4.3*. (**F**) Changes in *clec-126* after RNA interference with *col-41*. (**G**) Mechanism of action of *clec-126* with *col-41*. Compared with the control group. ^*^p < 0.05, ^**^p < 0.01, ^***^p < 0.001, ^****^p < 0.0001.

qPCR showed that overexpression of *clec-126* resulted in downregulation of *T02D1.7* and *nspf-3* and upregulation of *col-41* and *Y34F4.3* in *C. elegans* (Figure 5A). The analysis showed that *clec-126* was negatively correlated with *T02D1.7* (r = −0.2812, *P* = −0.5893) and *nspf-3* (r = −0.3859, *P* = −0.4499) and positively correlated with *col-41* (r = 0.9468, *P* = 0.042, significant) and *Y34F4.3* (r = 0.4589, *P* = 0.3600) (Figure 5B–5E). These observations were consistent with the qPCR results. After successful RNA interference with *col-41* in *C. elegans*, *clec-126* did not change significantly (*p* = 0.1336) (Figure 5F), indicating that *col-41* is a downstream gene of *clec-126* (Figure 5G).

## DISCUSSION

An increasing number of studies have shown that changes in the dopamine content of organisms vary with age [18]. The accumulation of dopamine can induce neuronal death and convert harmful substances into stable compounds in the brains of PD patients, thereby ameliorating neurodegenerative diseases and delaying the aging process [19, 20]. In recent studies of the aging process in wild-type C57BL/6 mice, it has been reported that these mice exhibited age-dependent dyskinesia, hindlimb abnormalities, and a decrease in the number of dopaminergic neurons in elderly subjects [21]. Thus, the loss of dopaminergic neurons associated with aging is one of the pathophysiological bases of aging.

Since Acein has been shown to be able to stimulate dopamine secretion in the brain [13], we decided to study its influence on aging. We selected the model organism *C. elegans*, which is classically used in aging research [22]. The dopamine content in *C. elegans* gradually decreases with the age. Acein was able to delay the decrease in dopamine levels and increase dopamine content in middle-aged and elderly *C. elegans* (Figure 1D). Dopamine may affect the growth and development, fertility [23] and the fitness [24] of *C. elegans*. Therefore, the number of body length, body width, number of broods, and frequency of body bending, head swing, and pharyngeal pumping were examined. We showed that Acein was able to improve the body length and width, number of broods, body flexion frequency, head swing and pharyngeal pumping of *C. elegans*, with a clear positive effect on aging and lifespan. Along with the study of antiaging activity, we examined the relationships with *clec-126*, a key gene regulating aging in *C. elegans,* identified by transcriptome sequencing. The *clec-126* gene encodes the C-type lectin domain protein [25] which as a negatively regulated gene, might be linked to body immunity [26]. To date and to our knowledge, no study has reported its role in aging. Our results suggest that *clec-126* expression gradually increases during aging in *C. elegans* in correlation with a decrease in dopamine content, thereby accelerating aging. In contrast, low *clec-126* expression correlates with increased dopamine content in *C. elegans*, thereby delaying aging. This suggests that *clec-126* overexpression may be an aging-related factor rather than a longevity-related factor. We showed that *clec-126* regulation did not affect dopamine changes in young *C. elegans* (Figures 3D, 4D), likely due to their well-established function of the dopaminergic system [27] in which neurons connecting the striatum can release dopamine normally [28], and probably plays a role in regulating various physiological functions of the central nervous system [29]. In aged *C. elegans*, Acein was able to increase dopamine levels and induce downregulation of *clec-126*, having a positive effect on aging and lifespan. Aging induced by high expression of *clec-126* in *C. elegans* is slowed down by treatment with Acein. Our results suggest that dopamine concentration is responsible for regulating *clec-126* expression. In humans, the lectin encoded by the orthologous gene *clec* is able to bind to glycans, which are receptors of virus-infected host cells, mediate microbial infections and negatively regulate immune function [30]. *Clec-126* as a “negative gene” affected the healthy lifespan of *C. elegans* and therefore might be involved in the immune response of *C. elegans*. We also demonstrated for the first time that *col-41* was a downstream gene of *clec-126* and that Acein promoted *col-41* expression after downregulating *clec-126*.

**Figure 6:**
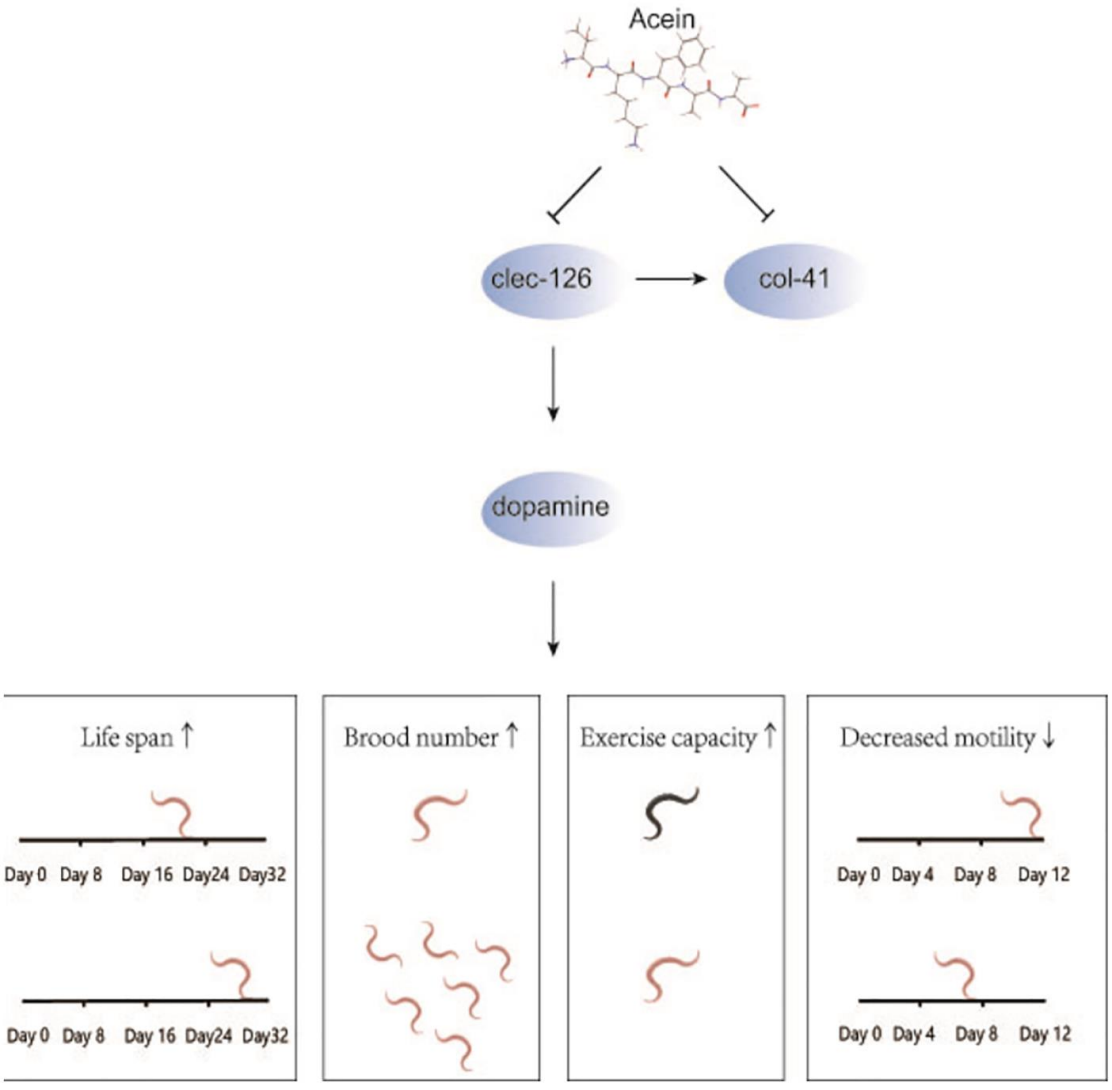
**Mechanistic diagram of the antiaging effect of Acein in the *clec-126* overexpressing *C. elegans* model.** Acein inhibits the expression of *clec-126* to increase dopamine secretion to prolong *C. elegans* lifespan, increase *C. elegans* brood number and motility, reduce motility decline, and delay *C. elegans* senescence. In addition, *col-41* was first found to be downstream of *clec-126,* and *clec-126* promotes *col-41* expression.

In conclusion, we have shown that Acein increases brood number and the fitness, ameliorates age-related functional decline and delays the aging process of *C. elegans* by regulating *clec-126* (Figure 6) to achieve the goal of prolonging healthy life. We have identified that Acein, most likely via its ability to stimulate dopamine secretion, can regulate the expression of *clec-126*. However, further studies are needed to determine the antiaging mechanism of Acein and whether *clec-126* can serve as a target for age-related pathologies in organisms.

## MATERIALS AND METHODS

### Synthesis and characterization of Acein

Acein was synthesized by the Fmoc protection strategy in SPPS with Wang resin. Fmoc-Pro-OH, Fmoc-Thr(OtBu)-OH, Fmoc-Lys(Boc)-OH, Fmoc-Phe-OH and Fmoc-Ala-OH were used (2.2 eq) and couplings were performed using HBTU (2-(1*H*-benzotriazol-1-yl)-1,1,3,3-tetramethyluronium hexafluorophosphate (HBTU) (2.2 eq) as coupling reagent in the presence of NMM (N-methylmorpholine) (4.4 eq) in a mixture of DMF-DCM (1:1) for 2 h. Each coupling was repeated twice. After coupling, the solvents were removed, and the resin was washed with DMF (3 times), methanol (2 times) and then DCM (2 times) for about 30 s each washing. Removal of the Fmoc protecting group was performed with a mixture of piperidine in DMF (20/80) for 10 min. Deprotection step was repeated twice, then washings were performed as described above. After completion of the couplings, the resin was treated with 5 ml/100 mg resin of the mixture trifuoroacetic acid/triisopropylsilane/water (TFA/TIPS/H_2_O) (95/2.5/2.5) for 3 h. The resin was filtered, rinsed twice with acetonitrile/water (1:1). The filtrate was collected and concentrated *in vacuo*. The residue was triturated in ethyl ether and centrifuged. The ethyl ether was removed. This was repeated three times. The residue was dissolved in a mixture of acetonitrile/water (1:1), freeze-dried in liquid nitrogen and lyophilized to yield the peptides as white powders. The crude peptide was purified by semi-preparative HPLC. The eluate with absorption value greater than 100 mV in the main peak was collected. The purity and the molecular weight of Acein were detected by analytical HPLC and LC-MS, respectively. The crude peptide purity of Acein was 82.4%, and the purity after purification was 97.5%. The physical properties of Acein are reported in Supplementary Table 3 and Supplementary Figure 1.

### Lifespan assay

Lifespan tests were performed at 20°C on *C. elegans* growth medium (NGM) plates inoculated with E. coli OP50. *C. elegans* were synchronized to the L1 stage, and when the larvae developed into the L3/L4 stage, 100 μL of a 5-FUDR solution (15.60 μg/mL) was instilled to inhibit oviposition and reproduction. The first day of lifespan tests was when the *C. elegans* developed into adults. Once daily, they were administered 10 nM Acein. The *C. elegans* status was observed daily until the death of all the nematodes. The daily number of deaths was recorded. The standard of judgment of death was when *C. elegans* were unresponsive to bright light or tapping on the plate, had no pharyngeal muscle movement at high magnification, and remained unresponsive to tapping of the head with a *C. elegans* picking needle.

### Growth and development test

In parallel, L4 stage larvae were collected with a *C. elegans* picking needle and transferred to a new NGM plate inoculated with OP50. Adult *C. elegans* were transferred to a new NGM plate after each egg laying until the end of the oviposition period. The number of larvae from all plates was photographed and the number of broods from ten *C. elegans* was recorded repeatedly in each group.

### Exercise capacity assay

Twenty adult *C. elegans* from each group were randomly selected and placed in new NGM medium. They were allowed to recover for 1 h. Then, an appropriate amount of M9 buffer was added to record both the number of times the *C. elegans* head swings from side to side and the number of body flexions for 30 s under an inverted microscope. The movement of the pharyngeal pump was amplified under an inverted microscope and more than ten *C. elegans* were videotaped for 30 s. Then, PotPlayer was used to slow down the video recording by 0.3 times to record the number of *C. elegans* pharyngeal pumps.

### Determination of changes in dopamine levels

A panspecies dopamine assay kit (Sango Biotech, China) was used to measure total dopamine content in approximately 500 *C. elegans*. Then, a BCA protein concentration test kit (Beyotime, China) was used to measure the total protein content. The relative level of dopamine secretion in *C. elegans* was expressed as the ratio of total dopamine content to total protein content, which was significantly comparable between different groups [31, 32].

### Quantitative real-time PCR

Sequencing of the transcriptome with Unique Molecular Identifiers (UMI) was performed on *C. elegans* samples. Differentially expressed genes were selected, ranked according to FC size. Genes with low reproducibility between parallel groups were excluded and genes with low expression were selected and then validated. Total RNA from cells was extracted by TRIzol reagent (Sangon Biotech, China) and transcribed into cDNA by a reverse transcription kit (Yisheng, China). cDNA was amplified by SYBR Green PCR Master Mix (Yisheng, China), qRT-PCR (ABI, USA) was performed using QuantStudio3, and GAPDH was used as an endogenous normalization control. The 2^−ΔΔCt^ method was used. The primers are shown in Table 1.

**Table 1:** Primer sequence information table.

**Target gene**	**Forward primer**	**Reverse primer**
*pmp-3*	ATCGTCTTCACTCGGATTGCCTTG	TCCTCTTCCTGCTCATCTCGTTCC
*gstk-2*	CAACGTATTCTTGTCGCTTCAC	TCACTCATCATCACCAACTCATC
*vit-2*	TTCACCGCTCATACCTTCTCAAGA	CTCATCAGATTGCTCCTCGTCTCT
*T02D1.7*	CATCTTCATCTCTTCCGTTCAAG	TTCTGGACTCATGTTGGATTCAC
*E02H9.9*	AGATTGGAGAGACTGATTGGATGGT	GTCGTGGATGATGAGCATGTAGATT
*clec-99*	CACATGCTGCCACTCAACAATG	CCTCTAACAGTCCTCGCCACAT
*F26D2.16*	GTCTCATGTCATTCCGCCATTCG	CCATCCTTCCGCATCCTTTGTATG
*nspf-3*	TCGCCACTGATGAATATGACC	TATGACGAATCTCCTCCTTTGC
*clec-197*	TCAAACAGTGAAGCATCTGGAAAGG	TCCACCTCCGTATCCTCCATAGT
*Y51A2D.8*	GCCTTCAACTTTGCCGTAATC	GCTCTTCTACAATCGTCCTCAAT
*Y67D8C.7*	TGGATGTTGAGGAAGAAGATATTGG	CCACCACCACCGTACTTGT
*clec-136*	GGTTCATCAAGCTGACAATGGT	TTCATCGTCTGTATAAGCCTGC
*linc-1*	ATGTTCCGTCGTGTCTTCGT	CAACTGGAGCCTTGATGGTAAC
*Y37F4.3*	TTATCAAGAACACCTGCTCCAAGTT	GCTCTCTGAATGGACTCAAGAATGT
*ZK1025.3*	CAACTTCTCACAATGGTCACACT	TCCACTAACTGCGTATCGGTAG
*col-41*	TCACAGCCACCAGTCAGAG	GATGGATAGCTTGGAGATGGATAG
*clec-126*	GCAGTTCTAGCCAGAGTTCCAGT	CCAGGCAACCTCCTCCTTGTT
*ZK856.4*	CAGACGGAAGAAGCAAGATGAAGT	TCGGAAACTGGGAACTCATCGT
*K10G6.9*	TGTGTTGTCTACTGGTATCTGGAAC	GCTTCCTCTGCTCATTGATGTACT
*Y34F4.3*	GCTGGTGCAAGTATCCAATGT	CGAGTGACAATCCGAACGAAT

### RNAi clec-126 in *C. elegans*

The HT115 strain expressing the dsRNA of *clec-126* was constructed and inoculated in NGM culture dishes containing IPTG. Under the induction of IPTG, strain HT115 produced dsRNA *clec-126* and *C. elegans* was able to obtain *clec-126 C. elegans* RNAi from the edible strain HT115.

### *Clec-126* overexpression in *C. elegans*

*Clec-126* cDNA expression was driven by the *dat-1* promoter. The pPD95.77 vector was digested with HindIII (AAGCTT) and Age I (ACCGGT) restriction enzymes to release the MCS sequence to obtain the linear vector. The fragments of the *dat-1* promoter and the *clec-126* sequence were amplified, and then Gibson assembly was carried out. The reporter plasmid pCFJ104 (5 ng/ml) and the target plasmid of the Pdat-1::clec-126::GFP::unc-54 3′UTR (20 ng/ml) were formulated into a mixture and then injected into wild-type N2 *C. elegans*. The primers used for PCR validation of *clec-126* were F: TATTGGACAGATGGCTCG; R: TGTCTTGTAGTTCCCGTCA.

### Stress capacity assay

When cultured L1 stage larvae of *C. elegans* reached L3/L4 stages, 15.60 μg/mL of 5-FUDR was added to inhibit egg laying and reproduction. After reaching adulthood, the *C. elegans* were treated daily with 10 nM Acein. On the 4th and 8th days, they were removed and placed in a 35°C constant temperature incubator for stress experiments. The life and death of *C. elegans* were recorded every 2 h until the death of the last *C. elegans*. The determination of death was the same as that in the lifespan experiment, which is described above.

### Motor decline assay

After daily treatment with 10 nM Acein, *C. elegans* were taken on the 4th, 8th and 12th days to detect their capacity for motor behavior including head swing and frequency of pharyngeal pump movements. The detection method was the same as that described for the determination of the movement capacity of *C. elegans*.

### Statistical analysis

Statistical analysis was performed using GraphPad Prism 8. Data are presented as the mean ± standard error of the mean (SEM). A Shapiro-Wilk test was used to determine the normal distribution of the data. Independent sample *t* tests were used for comparisons between two groups and one-way analysis of variance was used for comparisons between different groups. Survival curves were compared and *P* values were calculated using log-rank analysis (Mantel-Cox). A value of *p* < 0.05 indicates a statistically significant difference.

## Supplementary Materials

Supplementary Figure 1

Supplementary Tables
