# Supplementary material for: The peptide Acein promotes dopamine secretion through clec-126 to extend the lifespan of elderly C. elegans
Source: Aging (Albany NY). 2023 Dec 27;15(24):14651–65. doi: 10.18632/aging.205150 (PMC10781461; doi:10.18632/aging.205150)
Supplement: Supplementary Figure 1 [file aging-15-205150-s001.pdf]

# SUPPLEMENTARY FIGURE

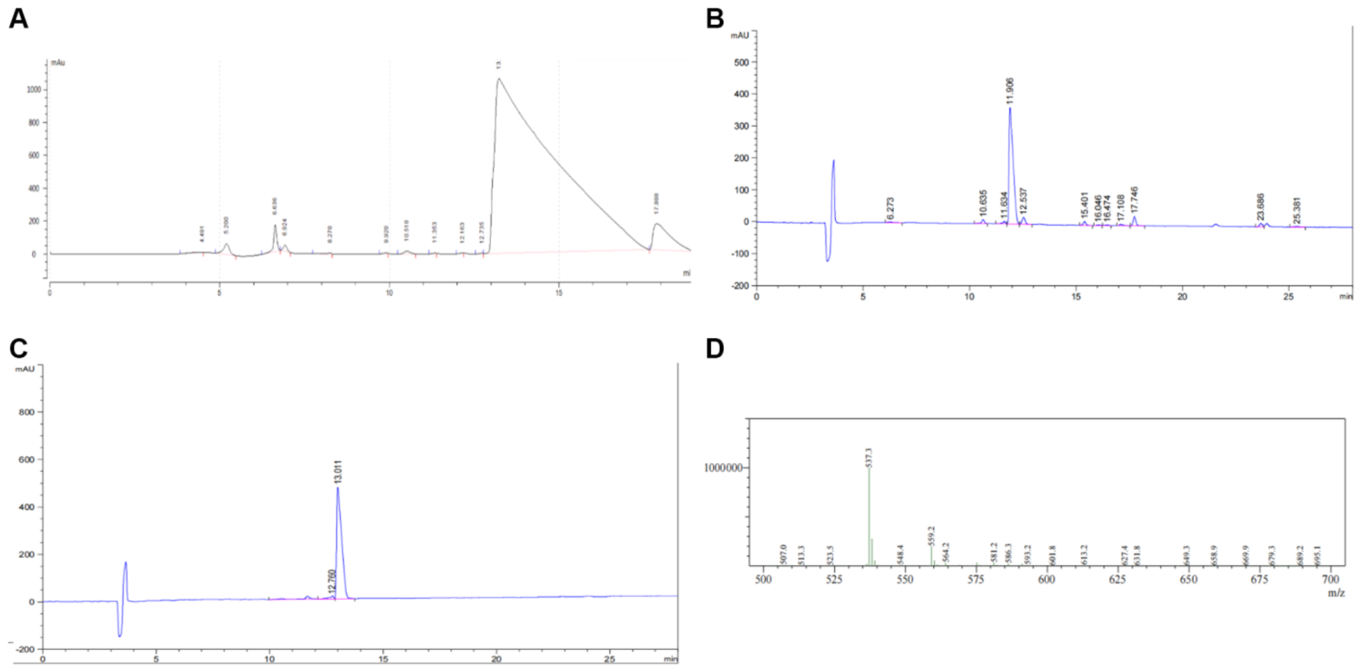


**Supplementary Figure 1. Synthesis and validation of Acein.** (**A**) Semi-preparative HPLC profile of Acein purification. (**B**) Crude HPLC profile of Acein. (**C**) HPLC profile of Acein. (**D**) Mass spectrometric of Acein.
