# Supplementary material for: The peptide Acein promotes dopamine secretion through clec-126 to extend the lifespan of elderly C. elegans
Source: Aging (Albany NY). 2023 Dec 27;15(24):14651–65. doi: 10.18632/aging.205150 (PMC10781461; doi:10.18632/aging.205150)
Supplement: Supplementary Tables [file aging-15-205150-s002.pdf]

SUPPLEMENTARY TABLES

Supplementary Table 1. Differentially expressed genes.

Gene name	Gene description	Gene length	Protein length	Ortholog	Function prediction
T02D1.7	Uncharacterized protein	225	74	none	none
nspf-3	Nematode Specific Peptide family, group F	264	87	none	none
col-41	Collagen composition	1450	428	none	Predicted to be a structural component of the stratum corneum, expressed in subcutaneous tissue and vulvar precursor cells.
clec-126	C-type lectin structural domain protein	1217	168	CLEC4G	Predicted to have carbohydrate-binding activity.
Y34F4.3	PAP-associated structural domain protein	4015	121	none	none

Supplementary Table 2. Information about *clec-126.*

Gene description	*clec-126*
Gene name	C-type lectin structural domain protein
Location	Chromosome II of *Caenorhabditis elegans*
Gene length	1217
Protein length	168
Protein location	–
Function	Predicted to have carbohydrate-binding activity.

Supplementary Table 3. Purity and yield of synthesized Acein.

	Acein
Purity of crude product (%)	82.40
Purity of pure product (%)	97.50
Amount of crude product (mg)	304.80
Amount of pure product (mg)	225.66
Yield of peptide purification (%)	74.04
